# Do SARS-CoV-2 Variants Differ in Their Neuropathogenicity?

**DOI:** 10.1128/mbio.02920-22

**Published:** 2023-01-18

**Authors:** Lisa Bauer, Debby van Riel

**Affiliations:** a Department of Viroscience, Erasmus MC, Rotterdam, The Netherlands

**Keywords:** neuropathogenesis, neuroinvasion, neurotropism, neurovirulence, CNS, astrocytes, SARS-CoV-2, Omicron, Delta, D614G, ACE2, NRP1

## Abstract

Neurological complications associated with severe acute respiratory syndrome coronavirus 2 (SARS-CoV-2) infections are a huge societal problem. Although the neuropathogenicity of SARS-CoV-2 is not yet fully understood, there is evidence that SARS-CoV-2 can invade and infect cells of the central nervous system. Kong et al. (https://doi.org/10.1128/mbio.02308-22) shows that the mechanism of virus entry into astrocytes in brain organoids and primary astrocytes differs from entry into respiratory epithelial cells. However, how SARS-CoV-2 enters susceptible CNS cells and whether there are differences among SARS-CoV-2 variants is still unclear. *In vivo* and *in vitro* models are useful to study these important questions and may reveal important differences among SARS-CoV-2 variants in their neuroinvasive, neurotropic, and neurovirulent potential. In this commentary we address how this study contributes to the understanding of the neuropathology of SARS-CoV-2 and its variants.

## COMMENTARY

Severe acute respiratory syndrome coronavirus 2 (SARS-CoV-2) infections are rarely associated with acute severe neurological complications like encephalitis. However, during the course of the pandemic it has become clear that SARS-CoV-2 infections are linked to a wide range of less severe neurological complications in both the acute and post-acute phase (1). These symptoms comprise, anosmia, sleep disorders, and cognitive deficits as well as psychiatric problems (2, 3) that can occur after both mild or severe SARS-CoV-2 infection. Neurological complications that last in the post-acute phase of the infection belong to the diverse spectrum of post-acute COVID-19 syndrome (PACS) or Long-COVID, which has a tremendous effect on the individual as well as on a societal level.

Since the start of the pandemic different SARS-CoV-2 variants have circulated that differ slightly in their ability to cause respiratory disease during the acute phase of the infection (4–6). Additionally, recent studies suggest that SARS-CoV-2 variants might also differ in their neuropathogenicity. For example, anosmia and Long-COVID are less frequently associated with Omicron infections compared to infections caused by D614G, Alpha, or Delta variants (7, 8), but no differences were observed in the risk of neurological and psychiatric outcomes between the Delta and Omicron waves (2). As the neuropathogenesis is difficult to study in humans—samples are limited, and often only available from the end-stage of disease—*in vitro* and *in vivo* models are pivotal in identifying differences in the neuroinvasive, neurotropic, and neurovirulent potential among SARS-CoV-2 variants. Furthermore, *in vitro* models can provide important insights into the virus-host cell interaction, as in the study of Kong et al. (9). Here, we will discuss the existing evidence for differences in the different aspects of the neuropathogenicity among SARS-CoV-2 variants and how future research can provide more insight.

## NEUROINVASIVENESS

Many studies have shown that SARS-CoV-2 has the capacity to invade the CNS. Viral RNA, proteins or infectious virus has been detected in postmortem CNS tissues from SARS-CoV-2 infected patients (10, 11) or experimentally inoculated hamsters (10, 12–14). In addition, intrathecal SARS-CoV-2 spike specific antibodies have been detected in the CSF of survivors, suggesting that virus entered the CNS (15–17). The olfactory nerve is an important route into the CNS for SARS-CoV-2 (10, 11, 18–20). Virus invasion via the olfactory nerve starts with virus infection of cells in the nasal olfactory mucosa, after which virus can travel along the nerve to the olfactory bulb. Recently, studies have shown that there are differences among SARS-CoV-2 variants in the ability to spread along the olfactory nerve to the olfactory bulb in experimentally inoculated K18-hACE2 mice and hamsters, where Omicron BA.1 variants did not enter the CNS as efficiently as D614G or the Delta variant (12, 21, 22). This reduced transmission of SARS-CoV-2 via the olfactory nerve to the CNS was associated with lower levels of virus replication within the olfactory mucosa and other parts of the respiratory tract. Additionally, fewer histological lesions were observed in the olfactory mucosa after Omicron inoculation compared to D614G or Delta inoculation (21–25). The fact that Omicron infections in humans are less frequently associated with anosmia fits with the reduced *in vivo* replication in the olfactory mucosa in animal models. Whether this is also related to a reduced neuroinvasive potential in humans and all different animal models is not comprehensively understood.

## NEUROTROPISM

SARS-CoV-2 is able to infect a wide range of neuronal cells, including olfactory sensory neurons in the olfactory mucosa, cortical neurons, dopaminergic neurons, astrocytes, and choroid plexus epithelial cells, although replication is often inefficient or abortive. *In vivo* large foci with infected cells are rarely detected (exception are K18-hACE2 mice) and *in vitro* efficient replication with virus release in the supernatant is restricted to choroid plexus epithelial cells (reviewed in references 26 and 27). An important factor for the cell tropism of a virus is its ability to attach and enter host cells. The entry process of different SARS-CoV-2 variants in CNS cells is poorly studied. SARS-CoV-2 host cell entry is a complex multistep process requiring receptor engagement and fusion protein activation of the Spike (S) protein (28). In infected cells, the polybasic cleavage site at the S1 and S2 junction of the S protein is cleaved by furin or furin-like proprotein convertases. Subsequently, the S2’ site needs proteolytic processing upon entering a new host cell. Interaction with the host cell receptor angiotensin converting enzyme 2 (ACE2) triggers a conformational change in the S protein that exposes the second cleavage site S2′. This second cleavage site can be cleaved by TMPRSS2 at the cell surface, facilitating fusion and release of the viral RNA at the cell membrane or by cathepsin L (CTSL) in the endosomal compartment (28).

While ACE2 is mainly expressed on type II pneumocytes in the lower respiratory tract, it is abundantly expressed on ciliated epithelial cells of the upper bronchus and nasal epithelium (29–31). However, in the CNS expression of ACE2 is limited and occurs predominantly on pericytes and choroid plexus epithelial cells (32, 33). Other proteins such as neuropilin 1 (NRP1), tyrosine-protein kinase receptor UFO (AXL), asialoglycoprotein receptor 1 (ASGR1), Kringle-containing protein marking the eye and the nose protein 1 (Kremen 1), dipeptidyl peptidase 4 (DPP4), basigin (CD147), lectins as well as sialic acids and heparan sulfate have been shown to serve as alternative receptors that may be utilized by SARS-CoV-2 to enter different cell types (34). Functional receptors for SARS-CoV-2 entry into CNS cells as well as the exact entry mechanism is unclear. In their latest study, Kong et al. showed that NRP1 but not ACE2 can function as an entry receptor for SARS-CoV-2 in primary astrocytes. siRNA knockdown revealed that besides NRP1 also CTSL, an endosomal protease enzyme with endopeptidase activity and tetrandrine (TPCN2), a nonselective endosomal two pore Ca^2+^-channel, reduced SARS-CoV-2 infection in primary astrocytes (9). However, even though NRP1 seemed essential for virus infection in this study, a previous study showed that SARS-CoV-2 D614G infection in primary and human inducible pluripotent stem cell derived (hPSC) cortical astrocytes, also lacking ACE2, was dependent on DPP4 and CD147 but not NRP1 (35). These studies suggest that ACE2 independent virus entry occurs in CNS cells. Currently it is not fully understood how SARS-CoV-2 enters the different cells in the CNS.

Emerging SARS-CoV-2 variants show multiple mutations in the S protein which can affect virus attachment to host cell receptors and membrane fusion essential for virus entry into the host cell. For example, S protein mutations that are present in the Alpha variant increases its replication in human ACE2-deficient cells compared to the D614G variant (36). Conversely, mutations in the S protein of Omicron BA.1 variant results in less efficient S1/S2 cleavage associated with a shift in cellular tropism away from TMPRSS2 expressing cells favoring the endosomal entry route compared to the D614G an Delta variant (37–39). If and how S protein mutations contribute to differences in infection efficiency or cell tropism of SARS-CoV-2 variants in CNS cells needs to be established. Recent *in vivo* and *in vitro* studies show that both primary human astrocytes (9) and hPSC derived human cortical neurons (12) are less susceptible for Omicron BA.1 compared to D614G and the Delta variant, suggesting differences in the neurotropism among SARS-CoV-2 variants. Using scalable *in vitro* models, like hPSC derived neural cultures or primary cells enable fast characterization of the neurotropism of emerging SARS-CoV-2 variants.

## NEUROVIRULENCE

The neurovirulence refers to the ability of a virus infection to cause CNS pathology that contributes to the development of clinical disease, which can be independent of the neuroinvasiveness or neurotropism of that virus. Evidence exists that the neuropathogenesis during a SARS-CoV-2 infection can be associated with systemic cytokine response, autoimmune antibodies, hypoxia, infection-associated coagulopathy, and/or virus infection in CNS cells (27, 40). For the latter SARS-CoV-2 seems to use a hit-and-run mechanism in the brain, where the virus enters during the acute phase of the infection after which the infection is aborted. Kong et al. showed that infection of brain organoids as well as primary astrocytes with SARS-CoV-2 D614G induced transcription of interferons and interferon stimulated genes as well as proinflammatory chemokines mounting an antiviral and proinflammatory response. At the same time genes important for maintaining synaptic plasticity were downregulated. This suggests that SARS-CoV-2 infection disrupts the neural homeostasis, creating an environment for promoting neuronal dysfunction and cytotoxicity (9).

Studies investigating differences in the neuropathology of SARS-CoV-2 variants are scarce. Since differences in the neuroinvasivenes and neurotropism of SARS-CoV-2 variants have been observed it is likely that there are also differences in the neurovirulence. We have recently shown that Omicron BA.1 inoculation of hPSC derived cortical neurons cocultured with astrocytes resulted in reduced antiviral and inflammatory responses (12). However, the study by Kong et al., showed similar responses in brain organoids inoculated with different SARS-CoV-2 variants, including Omicron (9). Future studies should reveal if there are differences in the neurovirulent potential of SARS-CoV-2 variants, and which underlying mechanisms contribute to these differences.

## CONCLUSION

We urgently need fundamental insight into the molecular mechanism of the neuropathogenesis of SARS-CoV-2 infections. Although different mechanisms can result in neuropathology, virus entry into the CNS and infection of CNS cells is an important one. Therefore, it is crucial to understand how SARS-CoV-2 can enter CNS cells, as it is clear that this differs from entry into respiratory cells. Unlike virus entry into respiratory epithelial cells, SARS-CoV-2 entry into CNS cells is seemingly not dependent on the canonical receptor ACE2 as suggested by Kong et al. and others (9, 35).

Neurological complications after SARS-CoV-2 infections are a huge societal problem so we need to acquire more knowledge on the underlying mechanisms of how SARS-CoV-2 impairs neural homeostasis, which will guide the development of effective intervention strategies. Recent studies have shown that hPSC-derived neural models, brain organoids or primary cells can be used to study the neurotropic and neurovirulent potential of SARS-CoV-2. As SARS-CoV-2 variants emerge quickly, we need to invest in these models and their scalability in order to determine the neurotropic and neurovirulent potential of these variants.
